# Detection of Resistance Mutations to Antivirals Oseltamivir and Zanamivir in Avian Influenza A Viruses Isolated from Wild Birds

**DOI:** 10.1371/journal.pone.0016028

**Published:** 2011-01-06

**Authors:** Goran Orozovic, Kanita Orozovic, Johan Lennerstrand, Björn Olsen

**Affiliations:** 1 Section for Zoonotic Ecology and Epidemiology, Linneaus University, Kalmar, Sweden; 2 Section of Zoonotic Ecology and Epidemiology, Department of Medical Sciences, Uppsala University, Uppsala, Sweden; 3 Section for Bioorganic and Biophysical Chemistry Laboratory, Linneaus University, Kalmar, Sweden; 4 Section of Clinical Virology, Department of Medical Sciences, Uppsala University, Uppsala, Sweden; University of Georgia, United States of America

## Abstract

The neuraminidase (NA) inhibitors oseltamivir and zanamivir are the first-line of defense against potentially fatal variants of influenza A pandemic strains. However, if resistant virus strains start to arise easily or at a high frequency, a new anti-influenza strategy will be necessary. This study aimed to investigate if and to what extent NA inhibitor–resistant mutants exist in the wild population of influenza A viruses that inhabit wild birds. NA sequences of all NA subtypes available from 5490 avian, 379 swine and 122 environmental isolates were extracted from NCBI databases. In addition, a dataset containing 230 virus isolates from mallard collected at Ottenby Bird Observatory (Öland, Sweden) was analyzed. Isolated NA RNA fragments from Ottenby were transformed to cDNA by RT-PCR, which was followed by sequencing. The analysis of genotypic profiles for NAs from both data sets in regard to antiviral resistance mutations was performed using bioinformatics tools. All 6221 sequences were scanned for oseltamivir- (I117V, E119V, D198N, I222V, H274Y, R292K, N294S and I314V) and zanamivir-related mutations (V116A, R118K, E119G/A/D, Q136K, D151E, R152K, R224K, E276D, R292K and R371K). Of the sequences from the avian NCBI dataset, 132 (2.4%) carried at least one, or in two cases even two and three, NA inhibitor resistance mutations. Swine and environmental isolates from the same data set had 18 (4.75%) and one (0.82%) mutant, respectively, with at least one mutation. The Ottenby sequences carried at least one mutation in 15 cases (6.52%). Therefore, resistant strains were more frequently found in Ottenby samples than in NCBI data sets. However, it is still uncertain if these mutations are the result of natural variations in the viruses or if they are induced by the selective pressure of xenobiotics (e.g., oseltamivir, zanamivir).

## Introduction

The scientific community has frequently expressed concern about the potential of influenza A virus to evolve into novel strains that can spread globally and induce pandemics [1]–[3]. These warnings were proven justified 2009 when the world experienced the last influenza A pandemic induced by strain H1N1, also known as swine influenza or new influenza. Fortunately, the new influenza was mild, as the viral infections in the majority of infected humans did not end with serious complications [4], [5]. All influenza A viruses originate from the avian influenza A viruses that naturally occur in waterfowl. The influenza genome encodes 11 proteins, of which one is non-structural. Avian influenza A is classified according the presence of two membrane proteins, hemagglutinin (HA) and neuraminidase (NA). There are 16 HA and nine NA identified subtypes, and the majority of subtypes (96 of 144) are found in the mallard duck (*Anas platyrhynchos*), assumed to be the major host and source of the influenza A viruses [6].

Vaccination is the most effective, cheapest and safest way to protect the majority of a population against influenza A, but vaccines can be difficult to rapidly produce in sufficient quantities during influenza pandemics. Thus, many countries stockpile two commercially available influenza A NA inhibitors, oseltamivir (Tamiflu®; active substance after oseltamivir processing in the liver is oseltamivir carboxylate (OC)) and zanamivir (Relenza®), as the main defenses against pandemic strains [7]–[9].

For both OC and zanamivir, high-level drug resistance is conferred by single or multiple nucleotide changes in the NA gene, as influenza A displays a high mutation rate and high viral replication. Long-term seasonal use of amantadine, a previously used antiviral of another class, has led to natural amantadine resistance in epidemic H3N2 and H1N1 viruses. Thus, human influenza A can develop resistance against both OC and zanamivir [10]–[18]. As both inhibitors bind to the catalytic site of NA, cross-resistance mutations are also found. Perhaps the most alarming news is the emergence of drug-resistant strains of the H5N1 subtype that cause high rates of mortality in humans [19]–[21]. The use of only OC and zanamivir as the first line of defense against pandemic strains has been disputed, and the need for new strategies or/and new antivirals has been proposed [22]–[24].

Furthermore, the use of both antivirals increases during seasonal influenza, especially during a pandemic, which results in higher concentrations of these substances in the environment. It is possible that wild birds (waterfowl) and, subsequently, influenza A viruses may come in contact with sewage water enriched with the antivirals. This may cause selection pressure on existing virus populations, and as a consequence, resistant mutants may be developed [25]–[29].

The aim of this study was to screen influenza A cDNA sequences for resistance mutations against the two existing neuraminidase inhibitors to determine the prevalence these mutations in the wild bird population infected with influenza A. In this investigation two data sets were used. The first data set was collected between 2002 and 2008 at Ottenby Bird Observatory and included 230 virus isolates from mallard. The second data set was obtained from the NCBI database and contained all bird, swine and environmental isolates of NAs of different subtypes.

## Methods

### Ethics Statement

Ethical approval for trapping, sampling, and keeping of birds was obtained from the Malmö/Lund Animal Research Ethics Board (M139-03).

### Virus sampling and q-PCR

Between 2005 and 2008 numerous cloacal samples from mallard ducks (*Anas platyrhynchos*) were collected using the cotton swab method. The sampling is a part of an ongoing surveillance at Ottenby Bird Observatory on the Swedish island Öland. The swabs were placed in 2-ml tubes with virus transport media [Hanks' Balanced Salt Solution containing 0.5% lactalbumin, 10% glycerol, 200 U/ml penicillin, 200 µg/ml streptomycin, 100 U/ml polymyxin B sulfate, 250 µg/ml gentamicin, and 50 U/ml nystatin (ICN, Zoetermeer, Netherlands)] that were immediately frozen at −70°C (at the latest, 30 min after sampling). The 100 µl of virus transport media was used for RNA extraction, which was performed using an EZ1 Virus Mini Kit (QIAGEN, Germantown, MD, USA) and extraction Biorobot EZ1 kit (QIAGEN), to yield a final volume of 75 µl of extracted RNA [30].

The presence of virus in the samples was confirmed using one-step q-PCR that targeted a conserved region of the avian influenza A matrix gene. Extracted RNA (2 µl) was used as template in the final reaction volume of 20 µl using a FastStart DNA Master SYBR Green I kit (Roche Diagnostics GmbH, Roche Applied Science, Mannheim, Germany). The amplification procedure was performed in a LightCycler 1.5 (Roche Diagnostics GmbH) under the following conditions: activation of polymerase for 10 min at 95°C and 43 cycles of 10 s at 95°C, 10 s at 60°C, and 10 s at 72°C. Finally, melting curve analysis was performed via a stepwise temperature increase from 65°C to 95°C, which identified the melting temperature of the reaction product [30].

### Virus growing and NA sequencing

All positive samples from q-PCR were grown in 11-day-old pathogen-free chicken eggs (allantoic fluid). Each sample was injected into two eggs and left at 37°C for 2 days, upon which the allantoic fluid was removed by syringe. The presence of virus was determined by hemagglutination assay using turkey erythrocytes. HA subtyping was performed by hemagglutination inhibition assay with subtype-specific hyperimmune rabbit sera [30].

The NA gene was sequenced to subtype the viruses. Total RNA was extracted from all hemagglutination-positive samples (High Pure RNA Isolation Kit; Roche Diagnostics GmbH, Germany) [31]. RT-PCR was done either according Hoffman [32] (110 isolates) or according to Orozovic [31] (120 isolates). In both cases, the PCR products were electrophoresed in 1.5% agarose and visualized with ethidium bromide [31]. The bands of 1,400 bp were cut out from the agarose gel, and the gel slices were purified using a QIAquick Gel Extraction Kit (Qiagen, Valencia, CA, USA). These 1,400-bp DNA fragments, representing NA genes, were sequenced by Macrogen (Seoul, Korea). The obtained sequence readings were assembled and processed using either DNASTAR® (DNASTAR, Inc., USA) or Vector NTI Advanced version 10.3.0 (Invitrogen. Co., USA). The whole sequences were aligned by BLAST, after which NA subtypes were identified [31].

### NA sequences analysis

Published literature was researched for mutations connected to both antivirals. NAs of all subtypes have eight conserved amino acids involved in the contact with substrates as well as in the function of the active site, and these are defined as catalytic residues: R118, D151, R152, R224, E276, R292, R371, and Y406. Ten additional amino acids, also well conserved, which are so-called framework residues (E119, R156, W178, S179, D198, 1222, E227, E277, N294, and E425), are involved in stabilization of the active site.

Nine mutations against OC (V116A, I117V, E119V, D198N, I222V, H274Y, R292K, N294S and I314V) and 10 mutations against zanamivir (V116A, R118K, E119G/A/D, Q136K, D151E/G/N, R152K, R224K, E276D, R292K and R371K) have been identified (N2 numbering; Table 1) [33]–[42]. Viruses with mutations R292K and V116A show resistance to both inhibitors. All mutations have been detected in human influenza A strains, with the exception of D198, which is found in an influenza B strain. As a reference, human N2 (accession number CAD35677) from the NCBI database was used.

**Table 1:** Overview on published oseltamivir and zanamivir related mutations.

Mutation	Inhibitor 1)	Type of residue 2)	Sensitivity in regard to NA subtype and acquisition 3)	
			Zanamivir	Oseltamivir
V116A	Z	F	(I) N1 ^a^	(R) N1 ^a^
I117V	O	F	(I) N1 ^a^, (S) N1 ^b^ *	(I) N1 ^a^, (I) N1 ^b^
R118K	Z	C	(nr) N2 ^a^	(nr) N2 ^a^
E119V	O	F	(S) N2 ^a^, (S) NB ^a^	(R) N2 ^a^, (R) NB ^a^, (R) N2 ^b^
E119G	Z	F	(R) NB ^a^, (R) N9 ^c^, (R) NB ^c^	(R) NB ^a^, (S) N9 ^c^
E119A/D	Z	F	(Nt/R) N2 ^a^, (R/R) NB ^a^, (R/Nt) N1 ^a^, (R/R) N2 ^c^	(Nt/S) N2 ^a^, (R/R) NB ^a^, (R/Nt) N1 ^a^, (I/S) N2 ^c^
Q136K	Z	F	(R) N1 ^a, c^	(S) N1 ^a, c^
D151E/G/N	Z	F	(S) N2 ^a^, (S/S/S) N1^c^	(LR) N2 ^a^, (S/S/S) N1^c^
R152K	Z	C	(S) NB ^a^, (S) N2 ^a^, (R) NB ^b^	(S) NB ^a^, (S) N2 ^a^, (R) NB ^b^
D198N	O	F	(R) NB ^b^	(R) NB ^b^
I222V	O	F	(S) N1 ^a^	(LR) N2 ^a^, (S) N1 ^a^
R224K	Z	C	(R) N2 ^a^	(R) N2 ^a^
H274Y	Z	F	(S) N2 ^a^, (S) N9 ^a^, (S) N1 ^b^	(S) N2 ^a^, (R) N9 ^a^, (R) N1 ^b^
E276D	Z	C	(R) N2 ^a^	(LR) N2 ^a^
R292K	O/Z	C	(R) N2 ^a^, (R) N2 ^c^	(R) N2 ^c^, (R) N2 ^a^, (R) N2 ^b^
N294S	O	F	(S) N1 ^a^, (Nt) N2 ^b^, (Nt) N1 ^b^	(R) N1 ^a^, (LR) N2 ^b^, (LR) N1 ^b^
I314V	O	-	(S) N1 ^b^ *	(I) N1 ^b^
R371K	Z	C	(R) N2 ^a^	(R) N2 ^a^

Mainly adopted from Ferraris and Lina [36].

1) Z - selected by zanamivir; O - selected by oseltamivir.

2) F - Framework residue; C - Catalytic residue.

3) Within bracket: R - resistant, I - intermediate, LR - low resistant, S - susceptible; nr - not recovered, Nt - not tested. Out of bracket: virus NA subtypes (N1 - 9 or B). Origin of mutants:

a) reverse genetic,

b) In clinic,

c) In vitro;

*- mutations included in double mutant.

**Figure 1:**
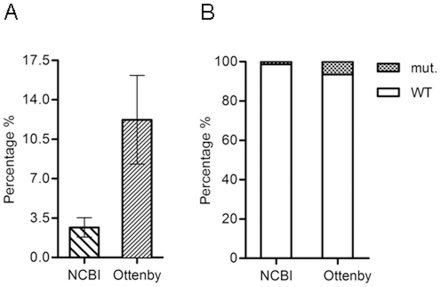
Comparison of mutant proportions from the NCBI and Ottenby databases carrying zanamivir and OC resistance mutations. A) The mean percentages of six replicates from the NCBI and five replicates from the Ottenby data set. All percentages represent subtypes containing mutant virus isolates. Subtypes without mutants are not represented in this analysis. The NCBI mean ± SEM percentage of mutants was 2.66±0.865; N = 6. The Ottenby mean ± SEM percentage of mutants was 12.22±3.931; N = 5. p<0.05, unpaired t-test. B) Frequencies of wild-type isolates in the NCBI and Ottenby data sets were 716 (98.76%) and 215 (93.48%) isolates, respectively. Frequencies of mutant isolates in the NCBI and Ottenby data sets were 9 (1.24%) and 15 (6.52%), respectively. In the NCBI data set, only viruses isolated from mallard were counted. Bars represent the percentage of wild-types and mutants in each data set. p<0.0001, chi-squared test.

The alignments using ClustalW were performed in BioEdit 7.0.8.0 which was also used to scan all of the above-mentioned mutations. The 230 mallard sequences from Ottenby as well as the 5490 avian, 379 swine and 122 environmental sequences obtained from the NCBI database (Table 2) were included in the analyses. Altogether, 6221 NA sequences were analyzed. The number of mutations for each subtype is expressed as a proportion of the total number analyzed sequences for that particular subtype (Table 2). The proportions of mutations for avian isolates of both NCBI and Ottenby sequences were pooled separately, which resulted in six replicates in the NCBI group and five replicates in the Ottenby group. To investigate if the proportion of mutants differed between the two data sets, the unpaired t-test was performed (Figure 1A).

**Table 2:** Summary of all virus isolates screened for antivirals mutations.

Isolate source	NA subtype	No. of isolates (x)	No. of mutants (y)	% (y/x)
Avian	N1	2133	57	2.67
	N2	1737	55	3.17
	N3	314	9	2.87
	N4	73	0	0
	N5	112	7	6.25
	N6	379	2	0.53
	N7	183	0	0
	N8	409	2	0.49
	N9	150	0	0
	All	5490	132	2.40
Swine	N1	169	0	0
	N2	203	18	8.87
	N3	4	0	0
	N6	1	0	0
	N7	1	0	0
	N8	1	0	0
	All	379	18	4.75
Environ.	N1	26	0	0
	N2	67	0	0
	N3	9	1	11.11
	N6	9	0	0
	N8	10	0	0
	N9	1	0	0
	All	122	1	0.82
Ottenby	N1	39	4	10.26
	N2	77	0	0
	N3	23	1	4.35
	N5	12	1	8.33
	N6	55	6	10.91
	N8	13	0	0
	N9	11	3	27,27
	All	230	15	6.52
	Total	6221	166	2,67

Also all sequences from mallard isolates (795 sequences) were extracted from the NCBI avian data set to form a new NCBI mallard group. Another group was made of the same Ottenby virus isolates from mallard. The frequency of wild-type isolates (no mutations) and mutants was organized as a contingency table and analyzed by chi-square test (Figure 1B). The null hypothesis was that the proportion of mutants was the same in both sources of sequences (NCBI vs. Ottenby).

## Results

### NCBI data set

The analyses of 5490 avian NCBI annotated sequences revealed 132 sequences carrying OC or zanamivir resistance mutations (Table 2 and Table S1). Eight OC-related mutations (I117V, E119V, D198N, I222V, H274Y, R292K, N294S and I314V) and six zanamivir-related mutations (V116A, R118K, E119G/A/D, R152K, R224K and R371K) were identified (Table 3). The majority of mutants were found in the N1 and N2 subtypes, which showed 57 (2.7%) and 55 (3.2%) mutants, respectively. The N5 subtype had the largest proportion of mutants (6.25%), while the N4, N7 and N9 subtypes did not have any mutants (Table 2). Subtype N1 showed one double mutant with I117V (OC-related) and E119G/A/D (zanamivir-related) mutations, and subtype N2 had one triple mutant with the zanamivir-related R118K and R152K and OC-related D198N mutations (Table 3). The most common mutation in N1 was the OC-related I117V, while the most common mutation for N2 was the OC-related I314V. H274Y (the most frequent resistance mutation in human influenza A) was seen in four isolates in N1 and one isolate in N2. Subtype N3 had three types of mutations, of which the OC-related I222V, which was found in five isolates, was the most frequent. Only one type of mutation was found in the N5 or N6 subtype. D198N was present in seven N5 isolates, and I222V was present in two N6 isolates. Subtype N8 had two different mutations: one V116A and one I117V (Table 3).

**Table 3:** Overview of antivirals mutations virus isolates from avian hosts (NCBI).

NA subtype	Mutation 1)	Type of residue 2)	No.
N1	(o) I117V; (z) E119G/A/D	F; F	1
	(o) I117V	F	36
	(z) R118K	C	1
	(o) E119V	F	1
	(z) E119G/A/D	F	2
	(o) I222V	F	5
	(o) H274Y	F	4
	(o) R292K	C	2
	(o) N294S	F	4
	(z) S/R371K	C	1
	All		57
N2	(z) R118K; (z) R152K; (o) D198N	C; C; F	1
	(z) V116A	F	1
	(z) E119G/A/D	F	5
	(z) R152K	C	2
	(z) D198N	F	1
	(o) I222V	F	1
	(z) R224K	C	2
	(z) H274Y	F	1
	(o) N294S	F	1
	(o) I314V		40
	All		55
N3	(z) V116A	F	1
	(o) I222V	F	5
	(o) L/I314V		3
	All		9
N5	(o) D198N	F	7
	All		7
N6	(o) I222V	F	2
	All		2
N8	(z) V116A	F	1
	(o) I117V	F	1
	All		2
	Total		132

1) (z) - zanamivir related mutations; (o) - OC related mutation.

2) C - catalytic residue; F - framework residue.

In the swine isolates, most of the subtypes did not have any NA inhibitor resistance mutations (i.e., subtypes N1, N3, N6, N8 and N9). The mutations were found only in the N2 subtype, which had 18 isolates (8.87% of the N2 subtype and 4.75% of all subtypes) with single mutations (Table 2 and Table S1). The majority of isolates had the OC-related I314V mutation, while the zanamivir-related Q136K mutation was found only in swine sample set (Tables 1, 2, 3). In total, 122 isolates from the environment and one N3 isolate (11.1% of subtype and 0.82 of total isolates; Table 2 and Table S1) had R152K (zanamivir-related) (Table 4).

**Table 4:** Overview of antivirals mutations in virus isolates from swine and environment (NCBI).

NA subtype	Mutation 1)	Type of residue 2)	No.
swine N2	(z) E119G/A/D	F	1
	(z) Q136K		1
	(o) D198N	F	1
	(z) R224K	C	1
	(o) I314V		14
	All		18
environ. N3	(z) R152K	C	1
	All		1
	Total		19

1) (z) - zanamivir related mutations; (o) - OC related mutation.

2) C - catalytic residue; F - framework residue.

### Ottenby data set

Mutants were found in 15 of 230 (6.52%) NA sequences from the Ottenby data set. Most of the mutants were found in the N6 subtype, which included six mutants (10.91%), followed by N1, with four (10.26%), and N9, with three mutants (27.27%). The N3 and N5 subtypes had only one mutant (4.35% and 8.33%, respectively) each, while the N2 and N8 subtypes did not show any mutants. Subtypes N4 and N7 were not part of this collection of sequences (Table 2). In the N1 subtype, I222V (OC-related) was found in one isolate. One isolate (68556) had only an R118K mutation, and isolate 68557 was a double mutant with R118K and D151N mutations. The fourth isolate (79959) was triple mutant with the zanamivir-related R118K, D151N and OC-related D198N mutations. This isolate had an additional R156K change in its sequence. However, this change is not related to resistance [39]. The NCBI collection of avian sequences had 2 out of 5991 (0.03%) mutants with more than a single mutation, while in the Ottenby collection the same type of mutants was found in 4 out of 230 (1.74%) isolates.

Mutants from both N3 and N5 subtypes had the R118K inhibitor resistance change. In all mutants belonging to the N6 subtype, R152K and the subtype-conserved D198N mutation were found. The OC-related mutation D198N has been observed only in influenza B virus. This study revealed that all NA sequences from subtypes N6, N7 and N9 had this change as a conserved feature. Two of the mutants from N9 carried one R118K mutation, and one isolate was a double mutant with R118K and D151N mutations. Furthermore, all isolates from this subtype had the conserved D198N change (Table 5).

**Table 5:** Overview of antivirals mutations in virus isolates from avian hosts (Ottenby).

NA subtype	Mutation 1)	Type of residue 2)	No.
N1	(z) R118K; (z) D151N; (o) D198N	C; C; F	1
	(z) R118K; (z) D151N	C; C	1
	(z) R118K	C	1
	(o) I222V	F	1
	All		4
N3	(z) R118K	C	1
	All		1
N5	(z) R118K; (z) R152K; (o) D198K	C; C; F	1
	All		1
N6	(z) R152K	C	6
	All		6
N9	(z) R118K	C	2
	(z) R118K; (z) D151N	C; C	1
	All		3
	Total		15

1) (z) - zanamivir related mutations; (o) - OC related mutation.

2) C - catalytic residue; F - framework residue.

### Statistical analyses

The unpaired t-test showed that the mean proportions of mutations in the NCBI (2.66%) and Ottenby data sets (12.22%) were different (p<0.05). Ottenby sequences had a higher proportion of mutants than NCBI sequences. In the succeeding analyses, frequencies of mutants within both data sets were compared. Here, only sequences belonging to mallards from NCBI data set (13.20% of all avian sequences) were included. The chi-square test showed that the frequencies of mutants in the two data sets (1.24% in NCBI and 6.52% in Ottenby) were different (p<0.0001), i.e., mutants were more frequent in the Ottenby data set.

## Discussion

### Resistance mutations related to subtypes

Resistance mutation patterns depend on the drug and the virus subtype. Additionally, some subtypes (e.g., influenza N2) are more sensitive to OC than to zanamivir, while the opposite is observed with other subtypes (e.g., N1) [14], [43].

In this study 6221 NA sequences were scanned for published anti-OC and anti-zanamivir mutations (Table 1). When the sequences from the NCBI database were compared with Ottenby sequences, some differences emerged. The subtypes N4 and N7 were absent in the Ottenby sequence collection, but these subtypes did not show mutations in the NCBI sequence collection. Subtypes N2 and N8 from the NCBI data set had isolates with mutations, while mutants were absent in the Ottenby N2 and N8 sequences. This could be explained by the small number of sequences for these subtypes in the Ottenby set (Table 2). In contrast, the N9 subtype collected at Ottenby had three mutants, while none was seen in the NCBI date set. In the case of the N9 subtype, factors such as host species difference and the location where the isolation was carried out could be important. However, it is also possible that the N9 subtype was more sensitive to selective forces, such as immunity, natural NA inhibitors [44] or even different xenobiotics distributed in the environment, including OC [25].

The largest proportion of mutants, 7 out of 112 (6.25%), in the NCBI database was in the N5 subtype, which could indicate that this subtype is the most prone to develop inhibitor resistance mutations. However, six isolates included the same species and were sampled at the same place (Table S1). These isolates were probably sampled at the same time form different individuals (same group of birds). Such relationships were observed in the many of the sequences that showed mutant genotypes, and information of this source is important when a detected mutation pattern is being interpreted.

Comparison of the subtypes present in both data sets showed that the Ottenby samples had a higher proportion of mutants than the samples from the NCBI database (Figure 1A). Again, this could simply have been an effect of a non-random grouping of data, which was characteristic for both data sets. All Ottenby isolates were collected between 2002 and 2008. The period when mutation-carrying NCBI isolates were collected varied depending on subtype. During 2002–2008, 93% and 84% of mutants were collected for subtypes N1 and N2, respectively, in the NCBI group. Only 25% of NCBI N3 isolates were from that period, whereas none of the N5, N6 or N8 isolates was. However, in the subsequent analyses, the time of data collection was ignored as a factor that potentially influenced the quantity of mutants.

The lowest proportion of mutants from the Ottenby data set was 4.35% for the N3 subtype, which was still higher than the proportions of mutants from all other avian subtypes from the NCBI data set, except for N5 (Table 2). The N5 subtype from Ottenby had 8.33% mutants (1 out of 11), which was higher than the same subtype from the NCBI data set. The highest proportion of mutants from Ottenby isolates was found in the N9 subtype, with 27.27% mutations (3 out of 11). The same subtype from the NCBI database did not show any mutations. Another remarkable difference between the two data sets was observed in the N6 subtype, where the proportion of mutants was almost 20 times higher in the Ottenby than the NCBI data set (Table 2).

These results indicate an increase of mutant frequency in the population of avian influenza A viruses in mallard duck from Ottenby. The reason for this can only be speculated. Given the migratory routes of mallard duck that include many populated areas in northern and western Europe [45], [46], it is appropriate to assume that mallards and consequently viruses could encounter water-borne OC or zanamivir more frequently than viruses in avian species collected from the NCBI data set. The possibility for recombination between avian and human virus strains already carrying mutations in the NA gene cannot be ruled out [3].

In swine, only the N2 subtype sequences contained mutants (8.87%), while in viruses isolated from the environment, only one mutant in the N3 subtype was observed. Among swine mutants, seven belong to H3N2 virus subtype, five to H1N2 and six to H9N2 virus subtype (Table S1). Two of the NA mutants (Q136K and D198N) from H3N2 subtypes have sequence similarity with human N2 which indicates that they probably originate from humans. The rest of NAs of H3N2 and all NAs from H1N1 virus subtype are most similar to swine N2 indicating origin from the same organism. On the other hand each NA mutant from H9N2 subtype shows similarity with NA isolated from chicken i.e. duck (A/Duck/Hong Kong/Y280/97) which implies avian origin. Adaptation of avian [47], [48] or human [49] N2 to the new host (swine) that led to changes in the swine N2 subtype might have provided conditions favoring a more frequent occurrence of antiviral resistance genotypes than in the avian N2 subtype (Table 2) [3], [5], [50], [51].

Regarding mutants isolated from environmental samples, only one with the zanamivir- related R152K mutation was found, dating from 2004 (Table 4 and Table S1). Altogether, 122 environmental NA sequences are available in the NCBI database, which may indicate difficulties in recovering virus genomic RNA from environmental samples or restricted efforts in doing so. The limited number of sequences from this analysis might explain why there were not more mutants within this group of sequences. The R152K mutation also occurred in the N2 subtype from the NCBI data set, as well as in the N6 subtype from the Ottenby data set. NA RNA from the environmental sample originating from Canada (Table S1) had the greatest similarity with chicken NA RNA that was also isolated in Canada. It is likely that the NA isolated from the environment originated from local poultry farms.

### Undetected resistance mutations in the study

Scanning of all avian NA sequences from the NCBI database showed that all known OC-related mutations were present in this data set [36], [52]. On the contrary, the zanamivir-related mutations Q136K [42], D151E and E276D [36], [40] were not observed in the same data set (Table 3).

The measured concentration maxima (Cmax) of OC [53], [54] and zanamivir [55] in the blood plasma can vary in the ranges of 1.4–1.9 µM and 0.05–0.43 µM, respectively. On the other hand, concentrations of both inhibitors that induce mutations *in vitro* are well above 1.0 µM [41], [56]. The peak concentration of OC in two studies from Japan was reportedly 0.001 µM [28], [29], but in studies from the UK and U.S., it was predicted to be as high as 0.05 µM and 0.1 µM, respectively [27]. Thus, it is apparent that in cases of induced antiviral resistance, the concentrations of NA inhibitors *in vitro* are well above the values detected or predicted in the environment. Even if OC or zanamivir had bioaccumulated in the waterfowl, it is likely that their concentrations would be much lower than OC concentrations known to select for resistant virus strains.

To summarize, a possible explanation for the lack of Q136K, D151E and E276D mutations in avian influenza A could be that virus variants with such changes in the NA gene are not part of the natural variation. However, even if they were, it is probable that selection pressure in the form of competition with other virus variants reduces the fitness of the virus so severely that these mutations do not develop.

### Detected primary and secondary mutations in this study

It has long been thought that reductions in viral fitness conferred by NA inhibitor resistance mutations would prevent transmission and the spread of resistance. However, recently NA inhibitor resistance has become apparent and has gradually spread amongst circulating seasonal influenza viruses worldwide. This could occur during prolonged treatment in, e.g., immunocompromised patients. Therefore, “permissive” secondary mutations emerge that compensate for the reduced fitness of the primary NA inhibitor resistance mutations [16], [18], [57].

#### In the NCBI data set

When the mutations found in the N1 subtype were categorized relative to two inhibitors (R292K excluded), 96% of all mutations were OC-related. Only one double mutant, with I117V and E119A mutations, was found in the same subtype. This mutant probably did not show a reduction in fitness, as it persisted in competition with other N1 versions that lacked those changes. The OC-related mutation I117V was the most frequent mutation, and it accounted for 63% of all mutants. It has been detected in NA of an H5N1 virus strain (A/Chicken/Indonesia/Wates/77/2005) that also had the I314V mutation, which made this strain a OC resistant double mutant I117V/I314V (Table 1) [34]. On the background of human strain H1N1 A/WSN/33, mutation I117V alone is sensitive to OC but weakly resistant to the NA inhibitor A-315675 [58]. The capability of I117V to reduce viral sensitivity to NA antivirals would probably depend on the presence and identity of secondary mutations [34]. I117V was, in 23 of 36 cases (64%), isolated at the same time, at the same place and from the same species (open-billed stork), which indicated that the same group of birds has been infected with the same virus subtype. The next most frequent mutations in the N1 subtype were I222V, H274Y and N294S (Table 3). I222V has been detected by reverse genetics (RG) in both N1 and N2 subtype, while H274Y and N294S have been found in human clinical isolates in the N1 and in the N1 and N2 subtypes, respectively (Table 1) [36]. The highly OC-resistant mutation H274Y is the most frequent mutation in human isolates of H1N1 viruses [16], [18] and has even been discovered in highly pathogenic avian H5N1 strains [20]. The ability of avian influenza A viruses to carry OC-resistant mutations was revealed in this sequence screening. This implies that these viruses possess enough fitness to cope with all the challenges imposed on them by the environments (different hosts, different types of open waters) in which they exist.

Of all mutations in the N2 subtype, the OC-related I314V mutation was dominant (73%) compared to the total proportion of all mutations (Table 3). This mutation has not been reported in the literature as a single mutation but only as a paired one with I117V [34]. Therefore, it is not clear whether it could alone influence changes in susceptibility to NA inhibitors. Still, its presence in viruses of wild populations could be potentially harmful if such viruses obtain additional mutation(s). One triple mutant was found within N2 sequences, carrying the R118K, R152K and D198N mutations. In this triple mutant, as in the case of the double N1 mutant, the fitness did not appear to be reduced dramatically, and it is possible that in both cases these changes in sequence could be compensatory regarding viral fitness [33].

Individual mutations R118K, E119V and S/R371K were specific for the N1 subtype, while V116A, R152K, R224K, and I314V were specific for the N2 subtype (Table 3). There were also differences in the frequencies of some mutations. I222V, H274Y and N294S were found in a higher number in the N1 subtype, while E119G/A/D was more frequent in the N2 subtype. The majority of mutations in the N1 subtype were OC-related, while most mutations in the N2 subtype were zanamivir-related (provided that I314V was not treated as a resistance-related mutation). In regards to the most frequent mutations for N1 (I117V) and N2 (I314V), it appears that they could be simply a natural form of NA, i.e., they were neutral mutations. The same was true for all N6, N7 and N9 subtype viruses that had D198N, which is otherwise related to OC resistance in influenza B viruses isolated in the clinic [36].

Of all sequences tested, only the avian N3 subtype had one isolate with the zanamivir-related V116A mutation [35], [58], and the rest of the mutations were I222V and S/I314V (Table 3). The N5 and N6 subtypes each had only one type of mutation. Moreover, the N6 and N8 subtypes had only two isolates with mutations. However, the low number of NA sequences for these subtypes compared to N1 and N2 (Table 2) made it difficult to draw any firm conclusions on the frequency and type of mutations.

Only five types of mutations were detected within swine NA sequences. The zanamivir-related Q136K mutation was only found here (Table 4). I314V was the most frequent mutation, and if its frequency is compared to frequency of avian N2, where the same mutation was also prevalent (Table 3), then this NA variant might have originated from avian NA, where it was likely a part of a normal gene pool variation for the N2 subtype. Furthermore, if I314V was ignored in both the avian and swine N2 subtype, then it appears that the rest of the mutations constituted 0.9% and 2.0% of the avian and swine N2 subtype populations, respectively. According to this, the swine N2 subtype was more receptive for antiviral resistance mutations than the avian N2 subtype.

#### In the Ottenby data set

In sequence collection from Ottenby (Table 5), only the N1 subtype had different mutation types, i.e., R118K, D151N, R156K, D198N and I222V. The rest of the subtypes had either the zanamivir-related R118K or R152K mutations only. The zanamivir-related mutation R118K obtained only by RG has not been possible to analyze by enzyme assay due to a complete lack of NA activity (Table 1) [40]. In the Ottenby data set R118K was present in all subtypes except in N6. In the N9 subtype from Ottenby, R118K showed the highest frequency of all mutations in all subtypes from both data sets. Interestingly virus H3N2 with the R118K mutation has been difficult to isolate from *in vitro* culture [40], but in the Ottenby set such mutants existed as a part of the natural population in mallard. The N1 subtype had one double and one triple mutant, with R118K/D151N and R118K/D151N/D198N mutations, respectively. The triple mutant had an additional mutation, R156K, but that mutation is not considered resistance-related. The presence of multiple mutations could be considered secondary compensatory mutations that work in synergy together with the first resistance mutation, which usually reduces the fitness of the virus. In the N1 subtype, half of the mutants carried multiple changes in NA sequences. If the relationship between the mutation frequencies and non-mutant frequencies were counted, then N1 had 18.00% of mutations instead of 10.26%. Such a high mutation rate could be an indication of the existence of additional selection forces not previously involved.

Comparisons of N1 mutations from both Ottenby and NCBI revealed some interesting details (Tables 1, 2 and 4). For example, the proportion of R118K mutations in Ottenby was 7.7% of the total number analyzed sequences for N1 subtype, while the corresponding mutation rate for the NCBI data set was only 0.04% of total sequences for the same subtype (Table 2). Two R118K mutants originated from successive years (2007–2008), which could indicate that this mutation was established in the wild virus population.

It is tempting to speculate that the wild virus population of the N1 subtype has gone through evolutionary changes driven by selective forces (antigenic drift, natural NA inhibitors or xenobiotics) and that it is not driven by evolutionary fidelity. If one such virus population carries such a resistance mutation, it could potentially be harmful if it obtains other resistance-related genetic shifts. Thus, contact between a wild bird virus population and a human strain is the only step necessary in this scenario. Such an event is possible either via direct transmission from birds to humans or via transmission involving a mixing vessel, such as pigs [3]. Furthermore, the mutation R118K (Table 5) has been experimentally induced (RG) only in the N2 subtype [36], and its instability seriously impacts viral fitness [40]. R118K was associated with N1, N3, N5 and N9 subtypes in the Ottenby data set. It could be that the spread of R118K was a result of a recombination event, i.e., R118K was transmitted from one NA subtype to the other. This would be possible when more virus strains infect the same host simultaneously, in this case the mallards. Mallards are birds that gather in high numbers at Ottenby. Still, not all of the birds are infected with the same virus strain at the same time, which significantly increases the chance that they could be infected with different virus strains.

In the Ottenby data set, besides the D198N found in the N1 triple mutant, I222V was another OC-related mutation. This mutation has been obtained by RG [36], [38] involving the N1 and N2 subtypes (Table 1), which did not show a resistant phenotype. However, in combination with mutation H274Y, its IC_50_ increased almost 2000 times for one H5N1 strain [41]. The mutation I222V was detected in one (2.56%) isolate in the N1 subtype from the Ottenby date as well as in five (0.23%) and one (0.06%) isolates in the N1 and N2 subtypes from the NCBI data, respectively. Thus, this “permissive and secondary” mutation was found in a much higher proportion in the Ottenby set.

Mutation D151N was present in two isolates from the N1 subtype as the second mutation besides R118K. This mutation was, until recently, only associated with RG experiments involving the human N2 subtype, where it showed resistance against zanamivir (Table 1) [36]. In a recent publication D151N was found alone or together with H274Y in human N1 isolates [38]. It did not influence sensitivity to any inhibitors alone, but in combination with H274Y it increased resistance to OC and to another NA inhibitor, peramivir. D151N can also result as adaptation to a new host, i.e., MDCK cells. The isolate 79959, with R118K and D151N mutations, also has R156K and D198N mutations. R156K is not related to inhibitor resistance (and therefore is not shown in Table 3 or 4), but D198N has been detected in influenza B isolates as an OC-resistance mutation [36]. D198N was also found in one avian isolate of N2 and all avian isolates of N5 from the NCBI data set (Table 3). The occurrence of D198N could be the result of either a compensatory change to an already present primary resistance mutation that reduced fitness or the result of a recombination with virus subtypes that had it as a conserved residue.

The N3 and N5 subtypes had the R118K mutation as the only mutation in the Ottenby set. The R118K mutation was not found in N3 from the NCBI data set. However, in the NCBI data set this mutation was detected in two avian isolates: one from the N1 and one from the N2 subtype (Tables 2 and 4). A similar trend was observed even in the case of the N5 subtype. In the NCBI data set the N5 subtype exclusively had the OC-related D198N mutation, while the N5 subtype from Ottenby had only the zanamivir-related R118K mutation.

The N6 subtype had six mutants (10.91%) with the catalytic residue change R152K. No such mutation was detected in the same subtype in the NCBI data set, which had two mutants (0.53%) with I222V (Tables 1 and 2). Interestingly the zanamivir-related mutation R152K, found in the bird NCBI data collection, was characteristic for the N2 subtype only, comprising 0.12% of the total number of sequences for the subtype.

Subtype N9 from the NCBI data set did not have any mutation at all. However, the same subtype from the Ottenby data set had the highest proportion of mutants (3 out of 11; 27.27%) relative to all subtypes of both data sets (Table 2). These three mutants had the same R118K change, which has been observed by RG on the N2 subtype [40]. However, a residue switch in one subtype does not necessarily relate to NA inhibitor resistance in another subtype. Still, this N9 catalytic residue change could indicate (as already mentioned in the case of the N1 subtype) that certain forces select for the accumulation of such resistance mutations in the NA gene.

The Ottenby isolates carrying the catalytic residue change R118K (N1, N3, N5 and N9) or R152K (N5 and N6) would be interesting to study in NA enzymatic inhibition assays, and such research is currently in progress in our laboratory. R118K is especially interesting because RG viruses carrying this mutation do not propagate well. There is also the possibility that these mutations were obtained as an adaptation in chicken eggs. However, it should be emphasized that almost all virus isolates in our study went through only one passage, so this event was less likely.

### Statistical analyses

NA subtypes that did not have any mutants were excluded from the total number of sequences. The test showed (Figure 1A) that the proportion of mutants was different between those two data sets (p<0.05). This analysis included all avian species from the NCBI data set, and it provided insight into how mean percentages of virus mutants were related in a more general fashion within these two data sets.

Within the analyzed NCBI sequences, the majority of isolates with mutations did not come from waterfowl (ducks, geese and swans) but from chicken, stork, turkey, quail, tern and herring. Ottenby samples were exclusively isolated from mallards. Transmission of influenza A from its natural host, mallard [59], to another bird species, such as chicken [51], or to another animal species in general [1], [3], [4], [50] could have led to changes in the NA gene that were the result of an adaptation to a new host. Thus, in order to avoid influence of NA gene changes arisen due to adaptation to a new host, all sequences from each NA subtype found only in mallards were extracted from NCBI data set and compared with Ottenby data set. The two data sets were put in a contingency table and analyzed by chi-squared test (Figure 1B), which gave the same result as the unpaired t-test. The frequency of mutations found in the Ottenby data set was higher than in the NCBI data set (p<0.001).

In summary, antiviral resistance–related mutations already exist in populations of avian influenza A viruses isolated from their natural hosts, i.e., mallard duck, other waterfowl as well as domestic poultry, and domestic swine. Therefore, several questions come into focus.

The first question is whether virus strains carrying resistance mutations are natural fluctuations of different virus versions [60]. Forces triggering such changes could be immune defense, natural NA inhibitors and adaptation to diverse transmission directions. Additionally, these changes could be a consequence of regulation of the balance between HA and NA activities subsequent to changes in HA affinity towards its cell receptor. Such an adaptation of NA as a response to changes in HA could lead to NA inhibitor resistance [61].

The second question is related to the need to investigate whether those mutations actually reduce NA sensibility to inhibitors. They could, in the light of differences in amino acids between avian and human virus strains, be neutral, i.e., they might not reduce sensibility to NA inhibitors. It would be interesting to investigate the naturally produced mutants by NA enzymatic inhibition assays to find out if they behave in the same way as their human counterparts. Such studies are currently in progress in our laboratory.

The third question concerns the source of transmissibility and antigenic shift of influenza A, which could be an important issue facing the next influenza outbreak. It is essential to investigate the transmission potential of those avian mutant strains to humans, as these strains might already be equipped with resistance against the currently used inhibitors OC and zanamivir. Therefore, the strategy of stockpiling influenza NA antivirals as a first line of defense against new pandemic strains could be endangered. In another study by us, preliminary results indicate that H1N1-infected mallards exposed to environmental concentrations of OC can develop the H274Y mutation (unpublished data).

The fourth and final question deals with the possibility of an increased frequency of NA mutations in the wild populations of viruses, as well as the emergence of novel and so far dormant subtypes (N9) in potentially harmful virus strains. Based on the results from the Ottenby data set, this possibility is not unreasonable (Figure 1A–B). It appears that certain selective forces have pushed the virus towards phenotypes that could be better equipped to infect a larger number of hosts. The threat of antiviral resistance in future influenza outbreaks warrants further exploration of alternative therapeutic strategies, e.g., new classes of drugs used in combination therapy with NA inhibitors [22], [23] or alternative technologies for faster production of influenza vaccine [62].

## Supporting Information

Table S1 List with mutants from NCBI dataset including protein ID, subtype, mutation, mutation sequence domain and strain name.
(XLS)
